# Family income and body mass index – what have we learned from China

**DOI:** 10.1186/s13561-016-0129-z

**Published:** 2016-11-25

**Authors:** Fafanyo Asiseh, Jianfeng Yao

**Affiliations:** College of Business and Economics, North Carolina Agricultural and Technical State University, 1601 E. Market Street, Greensboro, NC 27411 USA

**Keywords:** Family income, Body mass index, Obesity, Cross section, Panel Data China Health and Nutritional Survey

## Abstract

Obesity poses lots of health risks in both developing and developed countries. One thing that remains unclear is the relationship between family income and weight gain. This paper explores the relationship between family income and Body Mass Index (BMI) given variations in individual choice towards basic consumption and life quality improvement consumption as income increases. We use a nationally representative longitudinal data from China, the China Health and Nutrition Survey (CHNS), to estimate the relationship between income and weight gain. We conduct both cross sectional and panel data analysis to study the causal effects of family income on weight development. Unlike other literature that found inverse relationship between prevalence of obesity and family income in developing countries, in this paper, we find that BMI will first increase with family income at a decreasing rate, and then decrease which suggests that the group of middle class may suffer the high risk of being overweight and obese.

## Background

Obesity poses one of the greatest public health challenges facing both industrialized and developing nations [1]. We see particularly alarming trends in several parts of the world. Policymakers and the public have viewed with concern the dramatic growth in obesity that has taken place in developed countries over the last several decades, and in the recent years, in developing countries as well. In some developing countries, the obesity rate even bypasses the rate in the U.S. and keeps increasing. There is evidence of a strong link between being overweight or obese and chronic illnesses such as adverse metabolic effects on blood pressure, cholesterol, diabetes and cancer. Obesity also affects workplace productivity [2], employment [3] and the overall demand for and supply of health care [4]. In developing countries, rapid economic growth has also led to acceleration in nutritional transition in these economies which is contributing to the rate of obesity [5].

In China for example, evidence shows that from 1992 to 2002, there was a remarkable increase in obesity rates among various age groups, regions and gender [6]. Within the same period there was a reported massive growth in China’s economy. From the World Health Organization’s (WHO’s) body mass index (BMI) definition, the rate of overweight and obesity went up from 12.4 to 27.4% from 1991 to 2011 [7]. China was once considered to have one of the leanest populations, but it is fast catching up with the West in terms of the prevalence of overweight and obesity; disturbingly, this transition has occurred in a remarkably short time. With the economy growing rapidly in China and increasing transition within the population towards more Westernized behavior patterns, diseases related to being overweight are becoming increasingly burdensome and present urgent public health challenges, hence preventive strategies are required. Obesity is usually treated as a problem of public health or a misallocation of nutrition. In addition, it can also be regarded as a typical microeconomic problem because, unlike many other diseases, obesity can be avoided through individual behavioral change based on cost-benefit analysis. Naturally, people may rationally prefer to be under or over-weight in a medical sense, because weight results from personal tradeoffs and choices along such dimensions as occupation, leisure-time activity or inactivity, residence, and, of course, food intake. Given the variation in their choices about weight, being either fat or thin may be desirable from the individual’s standpoint as adhering to the norms of weight set by doctors and the public health community.

There are a number of reasons for the association between obesity and economic growth in many economies. Finkelstein, Ruhm [8] identified factors such as technological changes that lead to the lower food prices and increased food consumption as some of the factors that link economic growth and obesity. These factors also increased working hours, which is making more people eat in restaurants and fast food joints. Our study however is based on Blanchard’s model which shows that as wealth increases people may be more likely to spend initially on their material needs. However with time, they may move from just their material needs to healthier choices. This study aims at analyzing the relationship between income and BMI.

The theme of this study is to examine the associations of income with BMI given the variation in individual choices. We further test the hypothesis to investigate the relationship between income and BMI by using the data obtained through a large population based survey conducted in both urban and rural areas of regional Mainland China between 1989 and 2011 (CHNS). We analyze the correlation between adult BMI and family income in China looking at both static and dynamic effects. Our estimates are primarily derived using instrumental variable and fixed effect regressions, which handle the endogeneity and heterogeneity issues. We also consider the relationship between income at different intervals, quantiles and BMI. Given the structure of the data, the time effects are also taken into accounts.

Our study has a number of key contributions. This study is the first of its kind to analyze the relationship between family income and BMI using a large panel data set from China. Secondly, we explore how income effect varies by gender, age, and different BMI levels. Thirdly, our study investigates how family income and BMI change with time. Our main finding is that there is an inverted U shape between family income and BMI. The probability of being overweight first increases with age and then decreases as people get older. These results are found in both cross sectional and panel data analysis. For example, from a dynamic model, one more year of education increases the BMI by 0.079 units on average. Women’s body mass is roughly 0.491 higher than men’s body mass, and married women on average have a 0.204 lower BMI than single women. Other variables that had an effect on BMI included place of residence, age and education of the respondents.

## Literature review

Public health researchers have examined a series of factors in their quest to determine why obesity is increasing. At the most basic level, obesity is caused by chronic consumption of energy (calories) in excess of energy expended through metabolic and muscular activity [9]. Consequently, more physical activity, holding caloric intake constant, will result in a decrease in body weight. Studies using individual-level data have also identified race, age, and genetics as factors associated with obesity [10, 11]. Burke and Savage [12] show that the prevalence of obesity is significantly higher among African Americans. Kuczmarski [13] shows that the prevalence of obesity increases with age. Studies of twins have found that genes play a role in determining body mass index [14, 15].

However, a number of researchers have argued the current emphasis on determining the individually based risk factors that are relatively proximate causes of disease (e.g., diet and exercise) tells only part of the story [16–19]. Researchers must also consider the broader social factors that cause exposure to risk factors. Better understanding of how people are exposed to individually based risk factors may permit policy makers to design more effective (or cost-effective) means to combat diseases.

Much of the research on exposure to risk factors uses individual-level data to analyze the link between socioeconomic status, typically measured by either income or education, and morbidity or mortality. In the area of obesity, researchers have discovered that the prevalence of obesity is related to income. In a decade of economic growth and rising income, obesity has risen dramatically. This is puzzling when researchers have found that there is inconsistent relationship between income and obesity; most research on overweight and obesity draw on data from industrialized and high income countries, and results have been mixed. In a review of 144 obesity studies, Sobal and Stunkard [20] found that there is a strong inverse relationship between socioeconomic status (usually defined in terms of education and income) and obesity for women in developed societies the relation for men was weaker. Quintana Domeque and Villar [21] also explored the empirical relationship between family income and BMI in nine European Countries. Their findings suggest that the association is negative for women, but they also found no statistically significant relationship for men. They pointed out that the different relationship for men and women appears to be driven by the negative relationship for women between BMI and individual income from work. In support of these findings, Jeffery and French [22] argued that low socioeconomic status subjects lacked access to healthy foods, safe exercise and sound nutritional knowledge that caused their higher rates of obesity.

Other people argued different associations of BMI with income. Chou, Grossman [23] examined factors that may be responsible for the rapidly increasing prevalence rate of obesity. They employed the micro level data from the 1984–1999 Behavioral Risk Factor Surveillance System. They found a U-shaped effect of BMI on family income and hourly wage rates by age, gender, race, years of formal schooling completed, and marital status. However, their reported coefficients of income and income squared are relatively small. Later, Lakdawalla and Philipson [24] presented a dynamic theory of body weight and develop its implications. They argued that technological change has induced weight growth by making home- and market-production more sedentary and by lowering food prices through agricultural innovation. They also characterized how body weight varies with income. Their study presented descriptive empirical evidence that illustrates the inverted U-shaped relationship between body weight and income in U.S. males, suggesting the importance of secular trends in weight gain, which are consistent with the impacts of broad-based technological changes. Our study differs from theirs in that we take into account community- or individual fixed effect to examine the variation of BMI with income over time; i.e., genetic factor in the error term is associated with BMI and other variables such as education, entrepreneurial activity and income [25, 26].

We contribute to the literature studying the relationship between income and obesity in the following ways. First, previous researches lack a dynamic framework like what we use to explore the relationship between BMI and family income given variations in individual choices, and this may have significant impact on the results. This study hypothesizes that when income is low, overweight and obesity will start to develop and increase in severity. As income further rises, BMI will continue to increase but at a decreasing rate. Finally, BMI will decrease in income and will tend to stabilize (only at this time, they are roughly inversely related). That is why focusing on relatively low income country like China is more likely to let us see the above trend. Secondly, the model might be extended to examine the relationship between income and chronic symptoms like obesity. This dynamic model is inspired by Blanchard [27], but unlike him, we introduced a life quality improvement consumption in a closed economy, and our disease (death) rate is not constant, where the disease death rate can be determined by the life quality improvement consumption, which is more reasonable. Dynamic analysis and basic consumption will further give us a relationship between income and BMI. So, unlike other researchers, our model predicts a roughly inverted U-shaped relationship between BMI, or the prevalence of obesity, and family income. Thirdly, this paper is one of the first studies to use individual data from a developing country. The earlier literature has studied their association drawing mainly on data from U.S. and other industrialized countries.

## Methods

### Conceptual model

The theoretical framework of relationship between obesity and income is inspired by the model of Blanchard [27]. In Blanchard, the model is based on a closed economy with one final good which has two different consumption purposes: one is to satisfy the basic material needs, and two, is to improve the life quality such as disease prevention and body weight control. Within each time period, an individual rationally chooses the fraction of two kinds of consumptions. The finding is that when the income is low, the individual will mostly rely on the first kind of consumption, that is satisfy the basic needs and do not care much about life quality improvement. As a result, investment in disease or obesity control may not be enough. So, this low income individual will tend to gain weight and increase her BMI. As income gradually increases, she starts to pay more attention to the life quality improvement, and the investment in body weight control will correspondingly go up. However, achievement of disease prevention and body weight reduction should be lagged behind. Therefore, as she puts more resources into body weight control, the overweight problem will likely become more remarkable. Only after the treatment reaches a certain level, then body weight will be controlled properly, and with more income and investments, this individual’s BMI will gradually reduce. Later, as the control and treatment costs keep rising, the marginal net benefit of such control will gradually decrease. Thus, after BMI is reduced to certain level, this individual will transfer back the first kind of consumption, and her body weight will tend to stabilize. Therefore, as the income increases, the severity in overweight and obesity will first go up and go down (inverted-U shape).

### Theoretical framework

Consider a closed economy composed of an individual and a firm. Individual lives infinitely so that total labor in the economy is *L*_*t*_ = 1 at any time. Within each time period, the individual offers labor to the firm to let it produce the final good (being combined with capital). This final good has two purposes: one is to provide the basic consumption and two is to improve the quality of life. The individual’s current utility at time *t* is $u\left({c}_t\right)=\frac{c_t^{1-\theta }}{1-\theta }$ where *c*_*t*_ is the basic consumption and the parameter *θ* measures the degree of relative risk aversion that is implicit in the utility function (0 < *θ* <1). On the other hand, if this good is used to build health, it will improve the life quality.

Suppose an individual can always be threatened by diseases, we then use *p*_*t*_ to represent the probability the individual will get diseases at time *t*, so, from time *t* to *v*, the probability of not getting diseases is exp $\left(-{\int}_t^v{p}_sds\right)$. Diseases will bring negative effects on individual’s life. When someone is sick, the functionality of her body parts will be affected, and the life satisfaction at current state be discounted. So the larger the possibility of getting sick, the lower is the life satisfaction.

Although it is not avoidable that an individual may get diseases in any circumstance, it does not mean that nothing can be done about it. For example, an individual can utilize a part of resource such as the final good mentioned above to prevent diseases and improve the life quality, which will in turn improve the satisfaction towards current life condition. Assume the probability of getting a disease at time *t* is *p*_*t*_ = *p*(*x*_*t*_), where *x*_*t*_ is the quantity of the final good invested into disease control and life quality improvement. In the initial analysis, we put $p\left({x}_t\right)=\frac{1-\theta }{1+{e}^{x-\sigma }}$ where *σ* is exogenous, because after taking the first and second order condition of *p*(*x*_*t*_) with respect to *x*_*t*_, we will get: 1. *p* ' (*x*_*t*_) < 0: as investment of disease control increases, the probability of getting sick decreases. 2. When *x* < *σ*, *p* ' ' (*x*_*t*_) < 0: when investment is small, we won’t see an immediate large effect, and disease probability will slowly decrease. 3. When *x* > *σ*, *p* ' ' (*x*_*t*_) > 0: after the investment reaches a threshold *σ*, the disease precaution will slowly take effect.

From the time period t to infinity, the individual’s expected utility is

1 $$ {E}_tU={\int}_t^{\infty } \exp \left(-{\int}_t^v{p}_sds\right)\cdot {e}^{-\rho \left(v-t\right)}\frac{c_t^{1-\theta }}{1-\theta }dv $$

Here, (1) means only in the absence of disease, will an individual completely enjoy her life; when she is sick, she receives no satisfaction during the sick periods. For convenience, we transfer (1) into

2 $$ {E}_tU={\int}_t^{\infty }{e}^{-\left(p+\overline{p\left({x}_v\right)}\right)\left(v-t\right)}\frac{c_t^{1-\theta }}{1-\theta }dv $$

Where $\overline{\rho \left({x}_v\right)}$ is the average infection rate between time t and v, and

$$ \overline{\rho \left({x}_v\right)}\left(v-t\right)={\displaystyle {\int}_t^{\infty } \exp \left(-{\displaystyle {\int}_v^v{p}_s}ds\right)} $$

Assume the capital owned by the individual at time *v* is *a*_*v*_, and she rents capital to a firm to get interest *r*_*v*_ *a*_*v*_, and she also provides the firm with labor to get the wage rate *w*_*v*_. In the meantime, she uses her income for basic consumption, life quality improvement consumption and capital accumulation:

3 $$ {\overset{.}{a}}_v={r}_v{a}_v+{\pi}_v+{w}_v-{c}_v-{x}_v $$

Under this budget constraint, the individual maximizes her expected utility, and we will finally get the inter-temporal condition of the basic consumption (Euler Equation):

4 $$ \frac{{\overset{.}{c}}_t}{c_t}=\frac{1}{\theta}\left({r}_t-\rho -p\left({x}_t\right)\right) $$

And from first order conditions, we will also derive the relationship between basic consumption and life quality consumption *x*_*t*_:

5 $$ \frac{e^{x_t-\sigma }}{{\left(1+{e}^{x_t-\sigma}\right)}^2}{c}_t=1 $$

We also need to explore the behavior of the firm. During each time period, the firm rents capital from the individual and employs labor to produce. Since the individual has invested in life quality improvement and disease control, her productivity will be enhanced. However, this process is unstable and it is hard for a firm to measure it. So, the firm will treat this productivity improvement as exogenous enhancement and internalize it into the capital and labor:

6 $$ {k}_t^{\alpha }{x}_t^{1-\alpha }-{r}_t{k}_t-\updelta {k}_t-{w}_t $$

At equilibrium, where *a*_*t*_ = *k*_*t*_ after solving the maximization problem, the capital accumulation function becomes:

7 $$ {\overset{.}{k}}_t={k}_t^{\alpha }{x}_t^{1-\alpha }-{c}_t-\updelta {k}_t-{x}_t $$

Now, from (5), we will easily get the relationship between *c*_*t*_ and *x*_*t*_

8 $$ {c}_t={e}^{x_t-\sigma }+{e}^{-\left({x}_t-\sigma \right)}+2 $$

After taking first order condition of *c*_*t*_ with respect to *x*_*t*_, we will obtain:

9 $$ \frac{d{c}_t}{d{x}_t}=\left[{e}^{x_t-\sigma }-{e}^{-\left({x}_t-\sigma \right)}\right] $$

Where ${x}_t<\sigma,\ \frac{d{c}_t}{d{x}_t}<0;$ and when ${x}_t>\sigma,\ \frac{d{c}_t}{d{x}_t}>0$. That is to say as *x*_*t*_ increases, the fraction of *c*_*t*_ and *x*_*t*_ will first decrease then increase (U shaped relationship between *c*_*t*_ and *x*_*t*_ can be drawn).

Also, we may have the constraint that *c*_*t*_ + *x*_*t*_ ≤ *f*(*x*_*t*_) = *y*_*t*_. From the above relationship between *c*_t_ and *x*_*t*_, we learn that when the capital stock or output is small, both *c*_t_and *x*_*t*_ should be small, but *c*_t/_ *x*_*t*_ is large because when output is small, even if the individual allocates most resources to the “second” consumption *x*_*t*_, the disease probability may not be effectively reduced. So, the individual does not care much about the disease at this point and spends most of her income on the “first” consumption *c*_t_. After the output level is improved, the individual has the ability to allocate more resources to *x*_*t*_ and enough *x*_*t*_ will have an effect on disease control and health improvement. So the individual will tend to increase the investment into life quality improvement such as increase in household physical activity [28] which will gradually lead to the reduction in *c*_t/_ *x*_*t*_. Finally, when the production is large enough, a new problem arises; that is the diseasing marginal return of the investment in the disease or obesity control, which means even if the *x*_*t*_ is large enough, not much reduction in the disease severity will be observed, that is, *c*_t/_ *x*_*t*_ will increase again and the individual weighs more on basic consumption. Such relationship between *c*_t_ and *x*_*t*_ can be used to analyze the obesity and income level. Suppose the individual’s BMI is determined by *c*_*t*_, *x*_*t*_, and other variable vector *h*_*t*_:

$$ \frac{\partial B\left({c}_t\left({y}_t\right),{x}_t\left({y}_t\right),{h}_t\right)}{\partial {x}_t}>0\ \mathrm{and}\ \frac{\partial B\left({c}_t\left({y}_t\right),{x}_t\left({y}_t\right),{h}_t\right)}{\partial {x_t}^2}<0\;\mathrm{when}\frac{c_t}{x_t}>\theta\;\left({c}_t\ \mathrm{dominates}\ {x}_t\right); $$

$$ \frac{\partial B\left({c}_t\left({y}_t\right),{x}_t\left({y}_t\right),{h}_t\right)}{\partial {x}_t}<0\kern0.5em \mathrm{and}\ \frac{\partial B\left({c}_t\left({y}_t\right),{x}_t\left({y}_t\right),{h}_t\right)}{\partial {x_t}^2}<0\;\mathrm{when}\ \frac{c_t}{x_t}<\theta\;\left({x}_t\ \mathrm{dominates}\ {c}_t\right); $$

That is equivalent to: $\frac{\partial B\left({c}_t\left({y}_t\right),{x}_t\left({y}_t\right),{h}_t\right)}{\partial {y}_t}>0$, when $\frac{c_t}{x_t}>\theta$; $\frac{\partial B\left({c}_t\left({y}_t\right),{x}_t\left({y}_t\right),{h}_t\right)}{\partial {y}_t}<0$, when $\frac{c_t}{x_t}<\theta$.

When income is low, the individual does not care much about the effect brought by the obesity but mainly focuses on the basic consumption; so, as individual’s income increases, she will become more and more obese. However, when her income reaches a certain level, the problem of obesity will emerge and become remarkable enough to attract individual’s attention. At this time, the individual also has the ability of controlling this problem by increasing the second consumption. So, the speed of increase in overweight or obesity will gradually slow down. After the investment in obesity control reaches a threshold, say *σ*’, obesity will be controlled properly and its severity will gradually reduce. Finally, when the income is high enough, obesity is under some control, and marginal reduction of BMI or obesity severity is low, people will switch back to consume more *c*_*t*_ directly again, and the individual’s body weight will be stabilized.

### Empirical analyses

In our empirical study, we attempt to examine whether, as theoretical analysis predicted, adult BMI is correlated with family income in China, controlling for other factors. We begin with a discussion of several analyses that link income to BMI and obesity. We then specify the empirical test for each analysis.

### Cross-sectional framework

#### Linear regression and quantile regression model

Linear regression is a statistical tool used to model the relation between a set of predictor variables and a response variable. This model is able to estimate how, on average, family income affects BMI. While this model can address the question “is income important in determining BMI”, it cannot answer the important question “does income influence BMI differently for low BMI than for those who are overweight or obese?”, or put differently, is the relationship between BMI and income qualitatively equivalent to the relationship between obesity and income? A more comprehensive picture of the effect of the predictors on the response variable can be obtained by using quantile regression. Quantile regression models the relation between a set of predictor variables and specific percentiles (or quantiles) of the response variable. It specifies changes in the quantiles of the response. For example, a median regression of an individual’s BMI on her socioeconomic characteristics specifies the changes in the median BMI as a function of the predictors. The effect of income on median BMI can be compared to its effect on other quantiles of BMI. The quantile regression parameter estimates the change in a specified quantile of the response variable produced by a one unit change in the predictor variable. This allows comparing how some percentiles of the BMI may be more affected by certain individual characteristics than other percentiles. This is reflected in the change in size of the regression coefficient.

#### Interval regression model

Individual choice towards weight gain and obesity is different from normal consumption behavior such as the purchase of goods in the supermarket. The former can be defined as a “long term” behavior, and the results of choice will be revealed after a certain time period, while the latter is the instant choice decision and the choice consequence will be revealed within a short time period. This means that it is improper to use multinomial regression model which is used for describing the latter behavior to estimate the former case. The predictor variable and response variable of the former lack the clear-cut and immediate decision relationship compared to the latter. Interval regression model estimates an equation on the basis of data in which the dependent variable is only observed to fall in a certain interval or category on a continuous scale. The data are also censored in the usual sense in that both end intervals are assumed to be open-ended. The latent structure of the model to be considered is assumed to be given by ${y}_i={x}_i^{\hbox{'}}\beta +{u}_i$ (*i* = 1,…,N), where *y*_*i*_ is the unobserved dependent variable, *x*_*i*_ and *β* are both J x 1 vectors, the former being repressors and the latter unknown parameters. The *u*_*i*_ are assumed to be independent, identical and normally distributed random variables with zero mean and variance *σ* ^2^ and to be independent of *x*_*i*_. The conditional distribution of the unobserved dependent variable is given by ${y}_i\Big|{x}_i\ \to N\left({x}_i^{\hbox{'}}\beta,\ {\sigma}^2\right)$ *i* = 1,…,N. The observed information concerning the dependent variable is that it falls into a certain interval of the real line. The real line is divided into K intervals, the k-th being given by (*A*_*k*-*1*_, *Ak*) and these K intervals exhaust the real line. Thus *A*_0_ = − ∞ and *A*_*k*_ = + ∞, i.e., the first and K-th intervals are open-ended. The information on the dependent variable is which of these K intervals it falls into, i.e., an indicator variable *k*_*i*_ (1 ≤ *k*_*i*_ ≤ K) is observed for each *i*. We will use the following specification for the interval regression model:

10 $$ \beta X={\beta}_1{Y}_i+{\beta}_2{Y_i}^2+{\beta}_3AG{E}_i+{\beta}_4AG{E_i}^2+{\beta}_5{D}_i+{\varepsilon}_i $$

$$ P\left(BM{I}_{dis}=0\Big|X\right)=F\left({\alpha}_1-\beta X\right) $$

$$ P\left(BM{I}_{dis}=1\Big|X\right)=F\left({\alpha}_2-\beta X\right)-F\left({\alpha}_1-\beta X\right) $$

$$ P\left(BM{I}_{dis}=2\Big|X\right)=F\left({\alpha}_3-\beta X\right)-F\left({\alpha}_2-\beta X\right) $$

$$ P\left(BM{I}_{dis}=3\Big|X\right)=1-F\left({\alpha}_1-\beta X\right) $$

Where *α*_1_, *α*_2_ and *α*_3_ are thresholds of BMI categories defined by WHO, and *D*_*i*_ is a vector of demographic variables including highest education level attained, marital status, urban indicator, gender and region.

### Panel data analyses

Using longitudinal data, we will estimate the following specification:

11 $$ {W}_{it}={\beta}_0+{\beta}_1Yea{r}_t+{\beta}_2{Y}_{it}+{\beta}_3 Demo{g}_{it}+{\beta}_4Ag{e}_{it}+{\beta}_5Ag{e_{it}}^2+{\varepsilon}_{it} $$

The dependent variable is BMI adjusted for reporting error. This variable *W* plays the same role as in the theoretical framework. *Y*_*it*_ represents income, just as in the theory section, but in this regression *Y*_*it*_ will be included as a set of dummies indicating the quartile of the income distribution to which an individual belongs. There are two reasons for this. First, this specification allows for the inverted U-shaped relationship we predict. Second, if a person’s actual income is not well calculated or predicted, we can use her income category. *Year*_*t*_ represents a vector of year dummies. Next, we allow for weight to have an inverted U-shape in age: people gain weight as they approach middle age, but they begin to lose weight as they enter old age. This means that *β*_4_ should be positive, while *β*_5_ should be negative. Finally, we include a vector of demographic variables, *Demog*_*it*_, that contains highest education attained, and an indicator for being married with a spouse presently and an urban indicator. The regression specified above illustrates the conditional variation in weight across groups with different income status at a point in time (income quartile is always measured in the year of observation, relative to other respondents in that year). By estimating the empirical relationship between weight and various demographic characteristics, we can identify the growth in weight that resulted from demographic changes. The residual change here is attributed to technological change, in the tradition of economic growth-accounting. This relies upon the premise that changes in technology -by altering prices, incomes, and production technologies – cut across the population.

### Fixed effect model

Instead of examining variations in income and BMI across individuals at a point in time, we may estimate how changes in individual’s income over time influence changes in BMI over time. Here, if we assume fixed effects, we impose time-invariant individual effects that are possibly correlated with the regressors. Fixed effect model assists in controlling for unobserved heterogeneity when this heterogeneity is constant over time and correlated with independent variables. This constant can be removed from the data through differencing. The model set up is as follows:

12 $$ BM{I}_{it}={\beta}_0+{\beta}_1{Y}_{it}+{\beta}_2 Demo{g}_{it}+{\beta}_3Ag{e}_{it}+{\beta}_4Ag{e_{it}}^2+{u}_i+{\varepsilon}_{it} $$

Here, *u*_*i*_ is the unobserved individual effect, and $\varepsilon$_*it*_ is the time-variant error term. *u*_*i*_ could represent ability, genetics or historical factors that do not change over time. In this context, *u*_*i*_ is correlated with regressors (i.e., unobserved genetics factors are associated with income or demographic variables such as education.), and this unobserved heterogeneity may be purged by using fixed effect regression model.

Formally, we will get:

$BM{I}_{it}-\overline{BM{I}_i}=\left({X}_{it}-{\overline{X}}_i\right)\beta +\left({\varepsilon}_{it}-{\overline{\varepsilon}}_i\right)$ where *X*_*it*_ is a vector of predictor variables and ${\overline{X}}_i=\frac{1}{T}{\displaystyle {\sum}_{t=1}^T{X}_{it}}$ is the time average estimator. Therefore, the fixed effect estimator is:

$$ {\widehat{\beta}}_{FE}={\left({\displaystyle {\sum}_{i,t}^I{\widehat{x}}_{it}^{\hbox{'}}}\;{\widehat{x}}_{it}\right)}^{-1}{\displaystyle {\sum}_{i,t}^I{\widehat{x}}_{it}^{\hbox{'}}}\;{\widehat{y}}_{it} $$, where $$ {\widehat{x}}_{it}={X}_{it}-{\overline{X}}_i $$ and $$ {\widehat{y}}_{it}=BM{I}_{it}-{\overline{BMI}}_i $$

## Results

### Data and descriptive statistics

In this paper, the empirical work was based on the micro-level data retrieved from the China Health and Nutrition Survey (CHNS), which were collected by the Carolina Population Center (CPC) at the University of North Carolina at Chapel Hill, the Institute of Nutrition and Food Hygiene, and the Chinese Academy of Preventive Medicine. The study uses year 2000’s survey data for cross-sectional analysis and nine years’ longitudinal data for panel data analysis (1991, 1993, 1997, 2000, 2004, 2006, 2009 and 2011). The sample households were randomly drawn in eight provinces including Liaoning, Shandong, Jiangsu, Henan, Hubei, Hunan, Guangxi, and Guizhou. Two cities and four counties were sampled in each province. Four neighborhoods in each city, and one county-town in each neighborhood and three villages in each county, were then randomly selected. A neighborhood or village is defined as a community unit. Approximately 20 households were sampled per community. The CHNS data contain detailed information on household and individual characteristics as well as health-related information such as physical conditions, health behaviors and self-reported health status. The sample was restricted to men and women over the age of 18 for whom there exists a complete set of data on health and demographic variables (age, sex, marital status, education, family income, etc) were available. Since we needed to construct family income, we also exclude those with non-positive family and family income.

We now discuss a variety of measurement issues that need to be clarified before we present the estimation results. The main outcome variable BMI used to measure overweight and obesity, is based on self-reported data on height and weight. This allowed us to define the widely accepted BMI index indicator for each respondent. This index, defined as weight in kilograms divided by the square of height in meters (kg/m^2^), may also enable us to obtain an estimate of the prevalence of obesity. The WHO (1997) defines BMI below 18.5 kg/m^2^ as underweight, BMI of 18.5 to 24.9 kg/m^2^ as normal, BMI of 25 to 29.9 kg/m^2^ as overweight and a BMI of ≥30 kg/m^2^ as obese. Observations for those who lost their body parts and who were pregnant since their BMIs are not representative were also deleted.

To identify the family income, it is set up as the total household income inflated to 2011. We also control socio-demographic categories including age and age squared, highest education level attained, indicators for sex and marital status, family size, and year, rural and provincial indicators. The descriptive statistics for these variables are shown in Table 1. The average BMI for the population was 22.8, the BMI for women was a little larger (22.9) than that for men (22.6). On average the years of education was 17 years, with the minimum being 0 and the maximum being 22 years. Males made up 53% of the respondents. Majority of the respondents lived in rural areas (65%) and were married (88%). Table 2 provides the descriptive statistics for the panel data. The results are quiet similar to the year 2000 cross sectional data. Tables 8 and 9 in Appendix summarize the statistics of BMI, family income, and other variables before 2000 and after 2004. We observed positive trends of BMI, income and education, within which income has the fastest growth.

**Table 1:** Descriptive statistics of BMI, income and other variables in China, 2000 (*n* = 9,506 observations)

Variables	Mean	Standard deviation	Min	Max
Body Mass Index (BMI)				
Whole Sample	22.800	3.246	13.061	39.335
All Men	22.667	3.122	13.061	35.770
All Women	22.920	3.351	13.405	39.335
Cofactors				
HH Income (1000 2011CNY)	7.371	8.990	0	161.338
Highest Years of Education	6.769	4.175	0	18
Age	45.124	15.456	18	100.8
Marital Status (0 = not married, 1 = married)	0.868	0.338	0	1
Gender (0 = male, 1 = female)	0.524	0.499	0	1
Family size	3.920	1.457	1	11
Urban (0 = urban, 1 = rural)	0.658	0.474	0	1

**Table 2:** Descriptive statistics of BMI, income, and other variables in China, 1989 - 2011 (*n* = 80,230 observations)

Variables	Mean	Standard deviation	Min	Max
Body Mass Index (BMI)				
Whole Sample	22.725	3.293	8.954	39.792
All Men	22.622	3.175	8.954	39.792
All Women	22.817	3.393	9.033	39.751
Cofactors				
HH Income (1000 2011CNY)	10.642	17.398	0	658.472
Highest Years of Education	7.002	4.310	0	18
Age	45.828	15.896	18	100.8
Marital Status (0 = not married, 1 = married)	0.887	0.317	0	1
Gender (0 = male, 1 = female)	0.527	0.499	0	1
Family size	3.942	1.575	1	14
Urban (0 = urban, 1 = rural)	0.653	0.476	0	1

### Estimation results

The results for the regression are presented as follows. Table 3 shows the results for the linear regression measuring the effect of household income on adult BMI in 2000. To control for the endogeneity problem, family income is also instrumented by the family size. Family size is correlated with family income but not associated with error terms such as genetic factors; therefore, it satisfies the conditions for the instrumental variable. The first column shows the results for the whole sample, the second column shows that for men and the third column shows that for women. Table 4 presents results for quantile regressions and it shows the effect of family income on different quantiles of BMI. The first column is the 25^th^ percentile, the second column is the 50^th^ percentile and the third column is the 75^th^ percentile. In Table 5, we show the results of interval regressions with the effect of family income on various categories of BMI. The first column represents the whole sample the second column represents that for men and the third is that for women. Tables 6 and 7 are both results for the panel regressions. Table 6 shows regression results for family income on adult BMI between 1991 and 2011 and Table 7 shows the fixed effect model for the regression.

**Table 3:** Linear regressions measuring the effects of family income on adult BMI, 2000

Dependent Variable: BMI			
	Whole Sample	All Men	All Women
Income (1000 CNY)	0.836^c^ (0.094)	0.856^c^ (0.132)	0.640^c^ (0.146)
Income Squared	−0.035^c^ (0.008)	−0.036^c^ (0.011)	−0.030^b^ (0.012)
Constant	16.942^c^ (0.456)	17.590^c^ (0.602)	17.324^c^ (0.632)
Observation	7561	3717	3844
R-squared	0.185	0.181	0.179

Note: Numbers in parentheses are standard errors

^b^ and ^c^ represent significant level of 10, 5, and 1%

**Table 4:** Quantile Regressions Measuring the Effects of Family Income on Adult BMI, 2000

Dependent Variable: BMI			
	0.25	0.50	0.75
Income (1000 CNY)	0.809^c^ (0.091)	0.965^c^ (0.136)	0.874^c^ (0.154)
Income Squared	−0.032^c^ (0.007)	−0.033^c^ (0.012)	−0.033^c^ (0.014)
Constant	15.253^c^ (0.414)	15.854^c^ (0.593)	16.873^c^ (0.681)
Observation	9284	9284	9284
Pseudo R-squared	0.046	0.053	0.050

Note: Numbers in parentheses are standard errors

^a^, ^b^ and ^c^ represent significant level of 10, 5, and 1%

**Table 5:** Interval regressions measuring the effects of family income on categorical adult BMI, 2000

Dependent variable: BMI interval			
	Whole Sample	All Men	All Women
Income (1000 CNY)	0.522^c^ (0.090)	0.539^c^ (0.125)	0.352^b^ (0.141)
Income Squared	−0.024^c^ (0.008)	−0.021^b^ (0.010)	−0.025^b^ (0.012)
Constant	20.114^c^ (0.435)	20.769^c^ (0.615)	20.701^c^ (0.615)
Observation	7561	3717	3844

Note: Numbers in parentheses are standard errors

^b^ and ^c^ represent significant level of 10, 5, and 1%

**Table 6:** Regressions of specification (2) measuring the effects of family income on adult BMI over time, 1991-2011

Dependent variable: BMI			
	Total	Male	Female
Income Quartiles (1000 CNY)			
Bottom	0.639^c^ (0.139)	0.228 (0.195)	0.593^c^ (0.196)
Second	0.527^c^ (0.075)	0.367^c^ (0.103)	0.344^c^ (0.105)
Third	0.384^c^ (0.026)	0.302^c^ (0.033)	0.107^c^ (0.034)
Top	0.238^c^ (0.016)	0.026^a^ (0.015)	0.106^c^ (0.013)
Education	−0.079^c^ (0.010)	−0.026^a^ (0.015)	−0.130^c^ (0.014)
Marital Status	−0.078^c^ (0.021)	0.044 (0.033)	−0.204^c^ (0.029)
Gender	0.491^c^ (0.029)		
Year = 1991	−0.036 (0.058)	0.102 (0.080)	−0.159^b^ (0.084)
Year = 1993	0.016 (0.060)	0.167 (0.082)	−0.127 (0.086)
Year = 1997	0.211^c^ (0.062)	0.388^c^ (0.085)	0.055 (0.089)
Year = 2000	0.526^c^ (0.063)	0.661^c^ (0.086)	0.396^c^ (0.091)
Year = 2004	0.648^c^ (0.063)	0.802^c^ (0.087)	0.511^c^ (0.090)
Year = 2006	0.686^c^ (0.064)	0.869^c^ (0.088)	0.520^c^ (0.091)
Year = 2009	0.718^c^ (0.066)	0.892^c^ (0.091)	0.584^c^ (0.094)
Year = 2011	1.044^c^ (0.066)	1.178^c^ (0.091)	0.953^c^ (0.094)
Constant	17.584^c^ (0.156)	18.297^c^ (0.214)	18.365^c^ (0.211)
Observation	74416	35500	38916
R-squared	0.102	0.124	0.096

Note: Numbers in parentheses are standard errors

^a^, ^b^ and ^c^ represent significant level of 10, 5, and 1%

**Table 7:** Individual fixed effect regressions measuring the effects of family income on adult BMI over time, 1991–2011

Dependent variable: BMI			
Total	All	Male	Female
Income Quartiles (1000 CNY)			
Bottom	0.459^c^ (0.082)	0.734^c^ (0.117)	0.254^b^ (0.114)
Second	0.219^c^ (0.043)	0.137^b^ (0.060)	0.263^c^ (0.059)
Third	0.035^b^ (0.017)	0.038^b^ (0.021)	−0.005 (0.020)
Top	0.021^a^ (0.011)	−0.020^b^ (0.010)	0.025^c^ (0.009)
Constant	14.052^c^ (0.118)	13.619^c^ (0.161)	14.600^c^ (0.176)
Observation	74416	35500	38916

Note: Numbers in parentheses are standard errors

^a^, ^b^ and ^c^ represent significant level of 10, 5, and 1%

From Table 3, a 1000 CNY increase in family income causes adult BMI to increase by 0.836 units. Men showed a higher increase in BMI (0.856 units) than women (0.640 units). Income squared had a negative effect on the BMI in the whole sample, for both men and women. Once more this effect was more prevalent in men than women. Other confounders also had a significant effect on the adult BMI. We find that age, living in an urban area and being educated all had a significant effect on BMI.

To allow for the possibility of income varying across various BMI levels, a quantile regression was estimated at the 25^th^, 50^th^ and 75^th^ percentiles for the whole sample and the results are presented in Table 4. At the 25^th^ percentile of the BMI, a 1000 CNY increase in family income is associated with an increase of 0.809 BMI units. At the 50^th^ percentile, a 1000 CNY increase in family income is associated with an increase of 0.965 BMI units. At the 75^th^ percentile, a 1000 CNY increase in income is associated with an increase of 0.874 BMI units. Income squared had a negative significant effect on the various BMI levels. This explains the inverted U-shaped relationship between income and BMI. The quantile regression results suggest that the family income has consistent quadratic impact on the different BMI percentiles. The interval regression results are shown in Table 5, BMI category as defined by WHO is used for analysis. The outputs look very much like the ones from the linear regression model, and the estimation results are similar to those in Tables 3 and 4, which supports the theory that the risk of being overweight or obese, is associated with family income in an inverted U-shaped relationship. We also observed that the impact of income on BMI is smaller compared to the quantile and OLS regressions. Similar results were also observed when considering income squared and BMI.

As a means of checking the robustness we included time variables to the estimation. The results for males and females are presented in Table 6, which represents the analysis of BMI variation across different income distributions. We also observe an inverted U-shaped relationship for both males and females. We notice that as income level increases, the marginal effects of income on body mass accumulation tends to decrease because of the substitution between the demand for basic consumption and the demand for life quality consumption. Table 6, also suggests that weight growth may occur over time. From the coefficients of year dummies, we also observe that males accumulate body mass faster than females. Fixed effect regressions are used to control for the time-invariant heterogeneity and the results are shown in Table 7. Overall, our fixed effect estimators also demonstrate an inverted U-shaped relationship between BMI and household income over time.

## Discussion and conclusions

In this paper, we employ micro data from China to provide the theoretical examination and empirical test of the predictions linking household income to adult BMI using both cross-sectional and panel data analysis. We find some evidence supporting our predictions. Our results show an inverted-U shaped relationship between BMI and family income. Additional income brings about higher BMI and higher possibility of being overweight or obese for the poor than for the rich.

Furthermore, from the study, we observe that effect of income on BMI is more prevalent in the OLS regression that the other estimates that were done. Additionally, we find that relationship between income and BMI was more prevalent among those in the 50^th^ income percentiles. The BMI of males were more affected by family income than for women. Incorporating panel data in our study, we find that the relationship between BMI and income has been increasing over the years in China. Increased levels of BMI in general are troubling since this will also lead to an increase in chronic diseases among the population. Based on the cofactors we also find that the BMI increase was greatest in middle ages. This is very serious especially given the fact that middle age population plays a significant role in the growth and development of the economy.

While this study has its own limitations, it is among the first to provide evidence from a developing country on the nonlinear relationship between family income and BMI. Although the sample size is relatively small compared with the data in many U.S. studies, the set of CHNS data we have used is so far one of the best data sets used in studying income and BMI in the context of developing economies, and is probably the best Chinese data set. Finally, strictly speaking, our empirical tests are tests of correlations between family income and individual BMI. The causal link may not be established until more evidence becomes available regarding the intermediate mechanisms through which income affects obesity. However, we do find there is a strong relationship between family income and BMI.

## Appendix

**Table 8:** Descriptive statistics of bmi, income, and other variables in China, 1989–2000 (*n* = 39,952 observations)

Variables	Mean	Standard deviation	Min	Max
Body Mass Index (BMI)				
Whole Sample	22.091	2.997	13.061	39.627
All Men	21.910	2.836	13.061	37.687
All Women	22.256	3.128	13.071	39.627
Cofactors				
HH Income (1000 2011CNY)	5.302	6.379	0	178.293
Highest Years of Education	6.257	4.180	0	18
Age	41.658	15.383	18	100.8
Marital Status (0 = not married, 1 = married)	0.849	0.358	0	1
Gender (0 = male, 1 = female)	0.524	0.499	0	1
Family size	4.250	1.534	1	14
Urban (0 = urban, 1 = rural)	0.669	0.471	0	1

**Table 9:** Descriptive Statistics of BMI, Income, and Other Variables in China, 2004–2011 (*n* = 40,278 observations)

Variables	Mean	Standard deviation	Min	Max
Body Mass Index (BMI)				
Whole Sample	23.353	3.449	8.954	39.792
All Men	23.337	3.332	8.954	39.792
All Women	23.368	3.550	9.033	39.751
Cofactors				
HH Income (1000 2011CNY)	16.826	23.128	0	658.472
Highest Years of Education	7.678	4.316	0	18
Age	49.963	15.305	18	100
Marital Status (0 = not married, 1 = married)	0.924	0.263	0	1
Gender (0 = male, 1 = female)	0.530	0.499	0	1
Family size	3.634	1.555	1	13
Urban (0 = urban, 1 = rural)	0.636	0.481	0	1
